# Relationship between perceived threat of COVID‐19 and burnout among frontline nurses: A mediation analysis

**DOI:** 10.1002/brb3.2601

**Published:** 2022-05-04

**Authors:** Benard Gisilanbe Vetbuje, Panteha Farmanesh, Arman Sousan

**Affiliations:** ^1^ Business Management, Faculty of Business and Economics Girne American University, Girne Cyprus; ^2^ International Business Department, Girne American University, Girne Cyprus; ^3^ Faculty of Business and Economics Girne American University Girne Cyprus HRM Organizational Psychology

**Keywords:** burnout syndrome, diversity leaders, God attachment, perceived COVID‐19, religious coping strategy

## Abstract

**Background:**

Burnout of nurses during the Coronavirus of 2019 pandemic can end up extremely expensive for societies. It is found that positive religious coping (PRC) and a secure God attachment are effective for shielding against the adverse consequences of being exposed to stressful situations.

**Methods:**

This research explores the relationships among God attachment, religious coping, and burnout among nurses who are confronted with COVID‐19 as a perceived threat through a model based on the combination of attachment theory and the Job Demands‐Resources model. Analysis was done using SMART‐PLS.

**Results:**

The results reveal that perceived threat of COVID‐19 (PTC) positively correlates with burnout among nurses and that secure attachment to God and PRC can buffer this relationship, while insecure attachment to God, including anxious and avoidant attachment, along with negative religious coping positively mediates the relationship between PTC and burnout.

**Conclusion:**

Finally, this study suggests managerial implications of these findings for healthcare organizations and a recommendation for helping out staff to help them manage such threats and their attachment to God.

## 1 INTRODUCTION

The topic of burnout as a negative organizational outcome has been investigated throughout the organizational psychology literature. However, the focus of this study is on the medical environment, in particular burnout among nurses. The rise in measured compassion fatigue and turnover intentions among nurses have been reported in many former empirical studies before the COVID‐19 pandemic (e.g., Elliott, 2017; Gillman et al., 2015). Scholars explained their concerns regarding this organizational phenomenon and warned managers and policy‐makers about existing nursing shortfalls and their consequences (e.g., Donnelly, 2015; Elliott, 2017; Heinen et al., 2013).

During the pandemic, the situations have become worse regarding burnout in the medical environment, especially in areas related to the COVID‐19 (Ross, 2020). With the beginning of the 2021 vaccine rates, it will take approximately 7 years in order to retrieve normal life globally (Randall, 2021). Recent studies have provided statistical evidence regarding the association between nurses’ emotions and their burnout (e.g., Chen et al., 2021; Guixia & Hui, 2020; Hu et al., 2020). Lack of emotional wellness brings stress among healthcare workers, which leads to burnout and other negative consequences (e.g., Dincer & Inangil, 2020; Yıldırım & Solmaz, 2020; Zhang et al., 2020). High levels of emotional exhaustion among nurses fighting COVID‐19 have been reported in recent studies (e.g., Alharbi et al., 2020; Catton, 2020; Manzano García & Ayala Calvo, 2021). Religious coping has been investigated as one of the significant strategies for religious people in order to reduce their stress levels during pandemics (e.g., Chow et al., 2021; Fatima et al., 2020).

The significance of religion in workplaces has not been discussed sufficiently in the respective literature (Héliot et al., 2020; Schaeffer & Mattis, 2012). However, there are studies revealing a significant association between religiosity and different aspects of job attitudes (e.g., King & Williamson, 2005; Kutcher et al., 2010). Religion as a collection of beliefs has a crucial impact on individuals’ attitudes toward their environment, including workplaces, which leads to shaping their behaviors within their respective organizations (Farrukh et al., 2016). Therefore, it can be concluded that different orientations toward religion create diversity within an organization, which should not be neglected (Day, 2005; Rao, 2012).

Diversity management is considered an innovative practice capable of enhancing effectiveness and fairness (Alewell & Rastetter, 2020). Therefore, managers and organizational psychologists should pay attention to the concept of religion in the context of diversity management as a factor creating diverse groups with different attitudes and behaviors.

Religious people often seek security and resilience from their God in times of crisis (Brewer‐Smyth & Koenig, 2014; Faigin & Pargament, 2011; Ní Raghallaigh & Gilligan, 2010). Religion has been found to be an important socioemotional resource shielding staff against stressful situations (Byrne, 2011; Perera et al., 2018). However, there are very limited studies investigating the potential role of religion in burnout syndrome occurrence (Salmoirago‐Blotcher et al., 2016); in particular, this phenomenon needs empirical evidence in the nursing literature (Perera et al., 2018). Therefore, this study aims to contribute to the literature gap regarding the relationship between religion and psychological response to work stress, namely, burnout.

One of the concepts utilized in this study in order to investigate the role of religion for believers in the stressor‐burnout relationship is attachment to God (Kirkpatrick & Shaver, 1990), derived from classic attachment theory. According to this concept and its respective model, namely, attachment system activation proposed by Mikulincer and Shaver (2016), perceived health and social risk derived from confronting COVID‐19 (perceived threat of COVID‐19 [PTC]; Manzano García & Ayala Calvo, 2021) as the stressor of interest in this study would activate the attachment to God for religious individuals who seek proximity. If individuals benefit from a secure relationship with God, their attachment behavior would be constructive and manifested in positive religious coping (PRC): the reflection of a secure relationship with God (Hebert et al., 2009), which means without it, individuals suffer from an insecure relationship with God, and these insecure relationships can be categorized as anxious attachment to God (ANX): evaluation of God as a rejecting attachment figure (Mikulincer & Shaver, 2016), and avoidant attachment to God (AVO): evaluation of God as a distant attachment figure (Mikulincer & Shaver, 2016). The attachment behavior of people who are insecurely attached to God would be self‐destructive and manifested in negative religious coping (NRC): the reflection of a tenuous relationship with God (Hebert et al., 2009).

The other employed theory for exploring the role of religiosity in the transition of PTC into burnout is the Job Demands‐Resources (JD‐R) model proposed by Bakker and Demerouti (2007) and revised by Schaufeli and Taris (2014). According to this model, when the demands of a job outweigh the available job resources for an employee, the well‐being of that employee would be at risk of deterioration, which can lead to burnout. God, as a significant personal resource for believers (Gall & Bilodeau, 2021), can mitigate the adverse effects of job demands (Moon et al., 2020).

At the time of the COVID‐19 outbreak, when there are not easily available sources of support for frontline health workers, investigation of the effectiveness of PRC for believer nurses in reducing burnout has immense relevance. The aforementioned facts have become the underlying motivation for this study in order to investigate the causal mechanism of burnout through the lens of PTC, attachment to God and religious coping among Nigerian nurses dealing with COVID‐19. To the authors’ best knowledge, no study has investigated the effect of attachment to God and religious coping on burnout. This gap has gained more significance for further investigation due to the rise of burnout among health workers during the pandemic.

In order to explain the role of religion in the creation of burnout derived from the perceived threat of COVID‐19, this study is designed to incorporate attachment system activation and JD‐R models. To the best of the researchers' knowledge, the mentioned theoretical integration has never been proposed in the existing literature. In particular, we have investigated the mediation effects of attachment to God and religious coping in the relation between PTC and burnout among religious Nigerian nurses confronting COVID‐19.

## 2 LITERATURE REVIEW

Manzano García and Ayala Calvo (2021) explain that the perceived threat of COVID‐19 is about considering health and social risks while accepting them. They further delineate that the risk of infection by COVID‐19 (health risks), along with the risk of carrying this virus and spreading it to other people (social risks), are issues that nurses are confronting while treating COVID‐19 patients. Moreover, these nurses have accepted these risky situations by conducting their roles at the front line of medical services (Manzano García & Ayala Calvo, 2021).

There are a significant number of studies reporting the influence of PTC as a stressor on the attitudes and behavior of people during pandemics. For instance, in the medical environment, the situation is worse. Not only are healthcare workers opposed to the constant threat of the COVID‐19 virus, but they also witness death from COVID‐19 among patients and colleagues almost every day, which is a horrifying experience. Scarcity of essential protective equipment for nurses in underdeveloped countries has increased the risk of COVID‐19 contamination for nurses, leading to an increase in PTC (Irshad et al., 2020). Shahzad et al. (2020) implement a stressor–strain–outcome model and find that PTC has a positive association with psychological strains, including depression, anxiety and emotional exhaustion, explaining the agonistic behavior in paramedics confronting COVID‐19.

The effect of PTC as a stressor on burnout can be explained by the conservation of resources theory. According to the aforementioned theory, constant exposure to mismanaged stressors leads to the loss of emotional, psychological and physical resources, and burnout is defined as the deprivation of stated resources generated from chronic stress (Melamed et al., 1992; Shirom & Melamed, 2006). In accordance with the mentioned arguments, Manzano García and Ayala Calvo's (2021) found a positive correlation between PTC and burnout.

ANX and AVO, as insecure types of attachment to God, are found to have negative impacts on the cognition, emotions, and health of individuals. Insecure types of attachment to God have a negative correlation with forgiveness (Davis et al., 2008). Greater ANX is related to higher levels of religious anxiety (Fergus & Rowatt, 2014). Insecure attachment to God is positively correlated with alcohol use (Hernandez et al., 2010), eating disorder (Homan & Boyatzis, 2010), and health risk‐taking behaviors (Horton et al., 2012). Kelley and Chan (2012) provide empirical evidence implying negative associations for secure attachment to God with depression and grief. According to the mentioned definition of burnout as the impoverishment of cognitive, emotional, and physical resources, the authors expect a positive correlation between insecure types of attachment to God and burnout.

Religious coping is a set of responses by believers in stressful times, especially when there is a scarcity of effective resources (Oman & Reed, 1998; Smith et al., 2003). Pargament et al. (2011) categorized religious coping into positive and negative divisions according to the quality of the relationship between the believer and God:

- PRC is the fruit of a secure relationship with God, which can be manifested through the believer's trust in the existence of divine benevolent in everything, even harsh and bitter ones.
- NRC is the result of insecure relationships with God, which can be reflected through the believer's conflicts regarding finding meaning and purpose. In times of crisis, NRC is a bold reflection derived from beliefs regarding demonic interference, God's punishment or unavailability.

PRC contributes to well‐being while acting as a shield against adversity derived from stress and anxiety (Freitas et al., 2015; Pandey & Singh, 2019). Thomas and Barbato (2020) reported that PRC is negatively correlated with psychological disorders among Christian and Muslim residents of the United Arab Emirates at the time of the COVID‐19 outbreak. Haris and Tao (2021) provide empirical evidence implying that there is a negative association between PRC and burnout among nurses located in the southeastern United States.

On the other hand, NRC is associated with greater stress (Pirutinsky et al., 2020) and adverse outcomes such as depression, anxiety, and poor quality of life (Taheri‐Kharameh et al., 2016). Moreover, higher levels of NRC among people during the pandemic were related to greater feelings of loneliness (Yıldırım et al., 2020).

## 3 THEORETICAL LINKAGES AND CONCEPTUALIZATION OF THE MODEL

This study employed two theoretical schemes, namely, attachment theory and the JD‐R in order to explore the relationship between the perceived threat of COVID‐19 and burnout. Previous studies related to the nursing literature have found theoretical triangulation considerably beneficial for investigating and explaining the phenomena (e.g., Bennett, 1997), and its employment has been encouraged by scholars in this field (Halcomb & Andrew, 2005; Thurmond, 2001).

**FIGURE 1:**
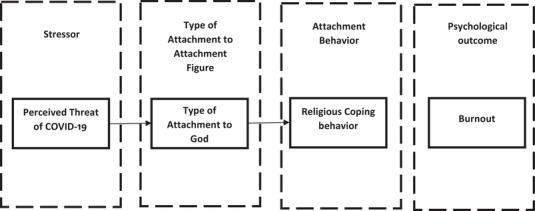
The role of religion in the transition of stressors into psychological outcomes

According to the attachment system activation model proposed by Mikulincer and Shaver (2016), perceived threat as a stressor activates the internal working model of individuals, leading to proximity‐seeking behaviors to attachment figures. Individuals cope with stressful situations through different strategies derived from the attachment type to their attachment figures. Thus, it can be said that religious people in exposure to stress seek proximity to God as the attachment figure, and depending on their attachment type to God, their attachment behavior becomes shaped. The aforementioned shaped attachment behaviors are manifested as religious coping behaviors, which influence the transition of stress into various psychological outcomes. Figure 1 illustrates the role of religion in the transition of stressors into psychological outcomes according to the attachment system activation model.

**FIGURE 2:**
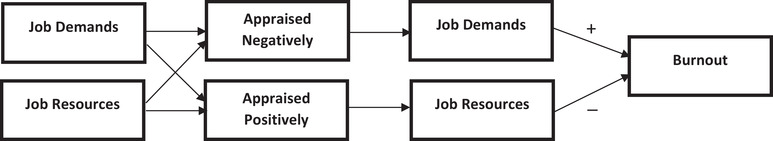
The effect of job demands and resources on burnout

According to the revised version of the JD‐R model (Schaufeli & Taris, 2014), which is aligned with the early JD‐R model, burnout is the result of high job demands, while the respective job resources are poorly low. However, the definition of job demands and resources has been changed in the revised model in such a way that only negatively appraised demands should be accounted as job demands and only positively appraised resources should be considered job resources. In the aforementioned redefinition, challenging demands are considered job resources due to the positive appraisal of the demands as challenging, whereas threatening resources are accounted as job demands due to the negative appraisal of the mentioned resources. Figure 2 demonstrates how job demands and resources influence burnout dependent on negative or positive appraisals.

According to Schaufeli and Taris (2014), perceived threat will increase job demand, which leads to burnout, while job and personal resources can mitigate this negative outcome. Availability of God has been found to be a significant personal resource for believers (Perera et al., 2018); thus, secure attachment to God, which is the reflection of perceived availability of God as attachment figure (Rowatt & Kirkpatrick, 2002), contributes to personal resources. On the other hand, insecure attachment to God, which is conceptualized as two types, namely, avoidant and anxious attachments, is about the unavailability or unresponsiveness of God in times of need (Mikulincer & Shaver, 2016), leading to the perception of resource loss that aggravates the problem of psychological distress (Heath et al., 2012).

The other employed theory for model conceptualization is based on attachment theory originally developed for parent–child relationships (Ainsworth, 1979; Bowlby, 1988) and later expanded into religious concepts (Pargament, 1997). Perceived threat in stressful situations triggers attachment system as a mental representation that comprised attachment figure and behavior explaining differences in human behaviors (Mikulincer & Shaver, 2016). Religious individuals seek protection and comfort from God as attachment figure when they perceive threat (Bradshaw, 2010).

Mikulincer and Shaver (2016) explain that insecure people are not sufficiently calm to employ effective practices to cope with stressful events; thus, PRC may not be the coping strategy employed by insecure people. Furthermore, Ryan et al. (2007) explain that felt security can shield individuals against the adverse consequences of stressful situations. Thus, secure attachment with God (lower levels of avoidant and anxious attachment) may buffer the generation of burnout derived from the perceived threat of COVID‐19. Therefore, the authors develop the following hypotheses:

Hypothesis 1a. AVO mediates the relationship between the perceived threat of COVID‐19 and burnout.
Hypothesis 1b. ANX mediates the relationship between the perceived threat of COVID‐19 and burnout.

Schaufeli and Taris (2014) redefine the demands and resources in a way that any negatively appraised resource should be considered as demand. PC is the result of a secured relationship with God, and NRC is the outcome of negative reappraisals of God's powers (Pargament et al., 2011). Thus, if believers appraise God negatively, God as a personal resource would be transitioned into demand, leading to exacerbation of burnout syndrome. Consequently, the authors formulate the following hypotheses:

Hypothesis 2a. PRC mediates the relationship between the perceived threat of COVID‐19 and burnout.
Hypothesis 2b. NRC mediates the relationship between the perceived threat of COVID‐19 and burnout.

Secure attachment to God as an attachment figure, which is reflected by the absence/low rates of anxious and avoidant attachments, will lead to mature and positive religious practices as attachment behavior appropriate for coping with stressful situations, whereas insecure attachment to God leads to a negative face of religiosity (Batson & Stocks, 2004; Pargament et al., 2011).

It has been found that the perceived threat of COVID‐19 has a significant influence on burnout (Manzano García & Ayala Calvo, 2021) and turnover intention (Irshad et al., 2020) among nurses. Burnout is a mental reaction caused by accumulated and mismanaged effects of stressors in confrontation with a perceived threat (Ceslowitz, 1989). On the other hand, it is found that positive psychological outcomes are predicted by secure attachment to God (e.g., Homan, 2014) and PRC (e.g., Ano & Vassoncelles, 2005), while negative psychological outcomes are positively related to insecure attachment to God (e.g., Parenteau et al., 2019) and NRC (e.g., Chow et al., 2021). Thus, the authors formulate the following hypotheses:

Hypothesis 3a. The relationship between the perceived threat of COVID‐19 and burnout is sequentially mediated by AVO and PRC.
Hypothesis 3b. The relationship between the perceived threat of COVID‐19 and burnout is sequentially mediated by ANX and NRC.
Hypothesis 4a. The relationship between the perceived threat of COVID‐19 and burnout is sequential

Mediated by AVO and NRC.

Hypothesis 4b. The relationship between the perceived threat of COVID‐19 and burnout is sequential

Mediated by ANX and PRC.

There are studies indicating significant differences in reported burnout according to marital status (e.g., Ifeagwazi, 2006; Ortega et al., 2018). Furthermore, it is found that core differences between Christianity and Islam faith systems have the potential to influence their attachment to God differently (Miner, 2012). Hence, we propose the following hypothesis:

Hypothesis 5. Statistically, significant differences exist in the structural coefficients for at least one among H1, H2, H3, and H4, considering the different groups in terms of marital status and religion types.

**FIGURE 3:**
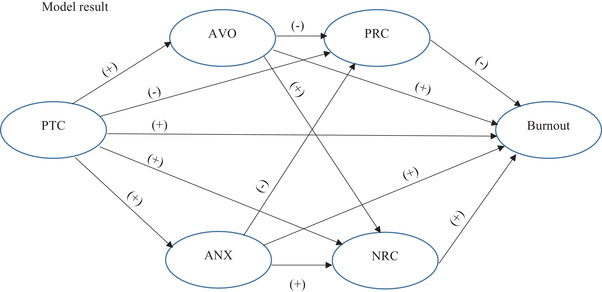
Theoretical model

Based on the aforementioned findings, the authors combined the hypothesized relationship of the perceived threat of COVID‐19 and burnout with attachment system activation and JD‐R models in order to explore the direct path between PTC and burnout and the indirect path of the mentioned variables through activation of attachment of God and its respective converged religious coping behavior. What the researchers expect from the mentioned theories and literature is that attachment to God and religious coping serially mediate the PTC and burnout relationship. The following theoretical model (Figure 3) presents the potential relationship between PTC, AVO, ANX, PRC, NRC, and burnout:

## 4 METHODOLOGY

### 4.1 Data collection process and sampling design

The recommended sample size was calculated by G*power for the proposed model, and the result number was 126 (1−*β *= 0.8, *f* ^2^ = 0.05). In addition, according to Hair et al. (2017a), the recommended sample size for our model (eight predictors, 1−*β* = 0.8, min *R* ^2^ = 0.10, *α* = 0.05) is 144. An online questionnaire survey was the instrument for collecting primary data for this quantitative study. Due to the pandemic, randomization was impossible for researchers at the time of conducting this research. Thus, this research have employed the combination of non‐probability sampling techniques, namely, convenience, snowball and purposive sampling (Etikan et al., 2016; Tongco, 2007). First, the authors contacted already‐known hospital managers as gatekeepers through the native Nigerian author in order to find potential participants and new gatekeepers (convenience and snowball sampling). As a matter of good etiquette, the gatekeepers’ permissions for data collection were granted, and in most cases, they made first contact on behalf of the researchers. The accessible nurses who agreed to participate in this research were further assessed according to devised selection criteria in order to extract eligible respondents (purposive sampling). The devised inclusion criteria were as follows: (i) nurses who were managing patients with suspected or confirmed COVID‐19 and (ii) nurses who had religious beliefs. In total, 346 responses were collected from eligible nurses, including 20 responses as a pilot test. Afterward, questionnaires were investigated for missing values and inattentive answers, and through the process, 24 responses were filtered out accordingly.

### 4.2 Respondents’ profile

The total number of responses employed for the investigation of the model was 302. Approximately 89% of respondents were female, approximately half of them were married (51%), and approximately half of them were Christian (50.6%). The average age was 31.3 (SD = 6.4), and the average job experience was 5.7 (SD = 4.9).

### 4.3 Questionnaire development and employed measurement scales

The anonymity of the respondents and confidentiality of their answers were informed to minimize common method bias (Podsakoff et al., 2003). In addition, to separate the measures, proximal separation was implemented by adding one filler regarding daily activities (Jordan & Troth, 2020). Finally, in order to investigate the common method bias rate in the collected data, collinearity tests were executed, and the variance inflation factors (1.6 ≤ VIF ≤ 2.7) were below the alarming threshold of 3.3 (Kock, 2015).

In order to measure the constructs of interest, this study employed scales that were validated in previous literature, and all the measures were anchored on a 7‐point Likert scale.

#### 4.3.1 Perceived threat of COVID‐19

The perception of respondents toward the COVID‐19 threat was measured by the scale developed in Manzano García and Ayala Calvo's (2021) study. This scale was used to measure this construct among nurses in recent pandemics.

#### 4.3.2 Attachment to God

Among pre‐validated constructs for measuring insecure types of attachments to God (anxious and avoidant), this study employed the Rowatt and Kirkpatrick (2002) scale with six items. Three items address avoidant attachment, with one reverse‐worded item and three items referring to the anxious attachment.

#### 4.3.3 Religious coping

Religious coping was measured with brief RCOPE that comprised positive and NRC as subscales (Pargament et al., 2011). Two subscales were treated as separate constructs to provide a better explanation for the hypothesized model.

#### 4.3.4 Burnout

The Shirom–Melamed Burnout Measure was used to measure burnout symptoms (Lerman et al., 1999; Melamed et al., 1999). This scale includes 14 items and three subdimensions, namely, physical fatigue, cognitive weariness, and emotional exhaustion. This construct is a reflective‐formative latent variable, which is schemed by a repeated indicator approach with mode B along with path weighting (J. M. Becker et al., 2012).

#### 4.3.5 Demographics as control and grouping variables

Since nursing is a female‐dominated sector, collecting a sufficient number of male respondents (according to the calculated sample size) to run a partial least squares‐multigroup analysis (PLS‐MGA) based on gender was not feasible for the authors. Therefore, gender as a potential predictor of burnout (Ortega et al., 2018; Purvanova & Muros, 2010) was used to control burnout. On the other hand, the dichotomization of continuous variables in controlled researches have been criticized due to findings implying a considerable reduction in statistical power (Maxwell & Delaney, 1993). Thus, job experience and age were not used for multiple group analysis; instead, the model was controlled by these potential demographic predictors (Brewer & Shapard, 2004). The control variables were treated as exogenous variables regressed on burnout as the dependent variable (T. E. Becker et al., 2016).

### 4.4 Analyses

The data analysis was conducted through PLS squares methods in SmartPLS version 3 software. First, we investigate the hypothesized model through the PLS‐SEM method for the following reasons: (a) the complexity of the structural model, (b) the exploratory nature of this study, (c) the relatively smaller required sample size, and (d) no need for normal distribution of data (Hair et al., 2019). Afterward, the moderation effect of grouping variables (marital status and religion type) on the structural model was investigated through PLS‐MGA in order to find significant differences between constructed groups (Hair et al., 2017b).

## 5 FINDINGS

### 5.1 Assessing the measurement model

The first step of this assessment concerns indicator loadings. Loadings as values vary between 0 and 1 should exceed the score 0.708 to be considered satisfactory (Hair et al., 2019). Loadings within a satisfactory level reveal that the construct is able to explain at least 50% of its indicators’ variance. All the loadings were examined, and they were above the cutoff value, showing the sufficient reliability of items.

The second step is to examine whether the scales employed in the current study are internally consistent. For this purpose, measures of Cronbach's alpha (Diamantopoulos et al., 2012), composite reliability (Jöreskog, 1971), and Rho A (Dijkstra & Henseler, 2015) were assessed, and the values were between satisfactory range of 0.7 and 0.9. Thus, it was assured that constructs are reliable in terms of internal consistency.

The third step involves establishing the convergent validity of constructs by examining the average variance extracted (AVE). The AVE of each construct is the mean of the square loadings of its items, and the acceptable score is more than 0.5, indicating that the construct is able to explain at least 50% of the variance in its respective indicators (Hair et al., 2017). The AVE scores for the constructs of the current study exceeded 0.5, enabling the research to proceed. Table 1 demonstrates the results of the first three steps of assessing the measurement model.

**TABLE 1:** Reliability and convergent validity of measures

Constructs	Subdimensions	Indicators	Outer loadings	Alpha	Rho A	CR	Average variance extracted
Perceived threat of COVID‐19 (PTC)		PTC1	0. 841	0.783	0.791	0.778	0.584
		PTC2	0.726				
		PTC3	0.899				
		PTC4	0.848				
Avoidant attachment to God (AVO)		AVO1	0.760	0.823	0.829	0.815	0.701
		AVO2	0.890				
		AVO3	0.888				
Anxious attachment to God (ANX)		ANX1	0.806	0.841	0.846	0.842	0.693
		ANX2	0.902				
		ANX3	0.844				
Positive religious coping (PRC)		PRC1	0.850	0.868	0.873	0.872	0.753
		PRC2	0.857				
		PRC3	0.875				
		PRC4	0.924				
		PRC5	0.751				
		PRC6	0.850				
		PRC7	0.885				
Negative religious coping (NRC)		NRC1	0.789	0.794	0.806	0.792	0.636
		NRC2	0.806				
		NRC3	0.835				
		NRC4	0.799				
		NRC5	0.833				
		NRC6	0.894				
		NRC7	0.729				
Burnout	Physical fatigue	PF1	0.725	0.809	0.814	0.805	0.573
		PF2	0.713				
		PF 3	0.791				
		PF 4	0.883				
		PF 5	0.795				
		PF 6	0.885				
	Cognitive weariness	CW1	0.779	0.824	0.827	0.825	0.734
		CW 2	0.846				
		CW 3	0.744				
		CW 4	0.752				
		CW 5	0.848				
	Emotional exhaustion	EE1	0.886	0.816	0.821	0.817	0.608
		EE2	0.864				
		EE3	0.843				

The fourth and final step for measurement model evaluation is to verify the discriminant validity of constructs, which means that constructs should distinctively operate in structural models based on the observations of the current study (Hair et al., 2019). The most reliable measure of discriminant validity is the heterotrait‐monotrait (HTMT) ratio with a conservative cutoff value of 0.85 (Henseler et al., 2015). Table 2 shows that the HTMT values of all employed constructs did not exceed the value of 0.85, establishing discriminant validity of the constructs. Since the reliability and validity of the measurement model are verified, the evaluation of the structural model can be conducted.

**TABLE 2:** Heterotrait‐monotrait ratio

	PTC	AVO	ANX	PRC	NRC	PF	CW
PTC							
AVO	0.609						
ANX	0.589	0.718					
PRC	0.674	0.616	0.836				
NRC	0.731	0.631	0.838	0.825			
PF	0.711	0.619	0.804	0.712	0.797		
CW	0.836	0.751	0.684	0.601	0.642	0.643	
EE	0.709	0.629	0.831	0.514	0.655	0.512	0.471

In order to assess the burnout as a reflective‐formative construct, (a) convergent validity, (b) VIF (collinearity), and (c) weight's significance and relevance have been tested (Sarstedt et al., 2019) and reported in Table 3. All the results meet a satisfactory level (Ramayah et al., 2018).

**TABLE 3:** Assessment of burnout as a reflective‐formative construct

Construct	Items	Convergent validity	Weights	Variance inflation factor	*t*‐statistics
Burnout	PF	0.737	0.504	2.377	7.412***
	CW		0.481	2.214	6.941***
	EE		0.428	1.905	6.273***

***p < .001.

### 5.2 Assessing structural model

The standardized root mean square residual ( = 0.033) and the normed fit index ( = 0.921) indicate an adequate model fit for the structural model (Henseler et al., 2014). In addition, the scores of the inner VIF were below 3, and thus, there was no concern about multicollinearity in order to proceed with the analyses. Finally, *R*‐squared (in‐sample predictive power) and *Q*‐squared (predictive relevance) scores as standard criteria for structural assessment (Hair et al., 2019) were calculated. PTC explains 16% and 18% of the total variances in AVO and ANX, respectively. In addition, the predictors of PRC and NRC account for 54% and 48% of their total variances, respectively. Finally, 72% of the total variance in burnout was explained by its antecedents. The scores of *Q*‐squared (AVO = 0.12; ANX = 0.13; PRC = 0.35; NRC = 0.31; Burnout = 0.43) were more than zero, indicating the adequate predictive relevance of predictors in the structural model (Henseler et al., 2009).

#### 5.2.1 Individual parameter estimates

**FIGURE 4:**
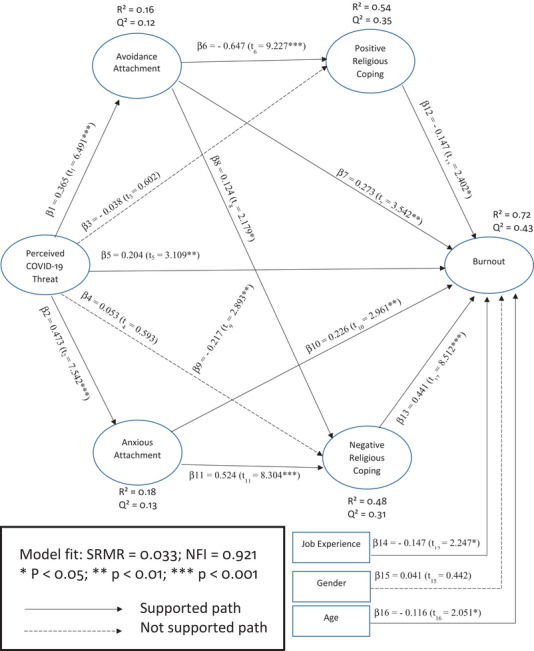
Path coefficients and significance from the results of partial least squares‐SEM

The results for PLS‐SEM estimations through the bootstrapping method with 5000 samples are presented in Figure 4. The statistical significance of path coefficients between variables was corrected for any bias through 95% confidence intervals. First, the results of this analysis reveal that there is a positive and significant relationship between PTC and AVO (*β*1 = 0.365; *t*1 = 6.49) and between PTC and ANX (*β*2 = 0.473; *t*2 = 7.542). Second, the results show that there was no significant relationship between PTC and PRC (*β*3 = −0.038; *t*3 = 0.602) and also between PTC and NRC (*β*4 = 0.053; *t*4 = 0.593). Third, the findings indicate that there is a positive and significant relationship between PTC and burnout (*β*5 = 0.204; *t*5 = 3.109). Fourth, the results demonstrate that there is a significant and negative relationship between AVO and PRC (*β*6 = −0.647; *t*6 = 9.227), a positive and significant relationship between AVO and burnout (*β*7 = 0.273; *t*7 = 3.542) and a positive and significant relationship between AVO and NRC (*β*8 = 0.124; *t*8 = 2.179). Fifth, the findings show that there is a negative and significant relationship between ANX and PRC (*β*9 = −0.226; *t*9 = 2.961), a positive and significant relationship between ANX and burnout (*β*10 = 0.226; *t*10 = 2.961), and a positive and significant relationship between ANX and NRC (*β*11 = 0.524; *t*11 = 8.304). Finally, there is a negative and significant relationship between PRC and burnout (*β*12 = −0.147; *t*12 = 2.402) and a positive and significant relationship between NRC and burnout (*β*13 = 0.441; *t*12 = 8.512).

#### 5.2.2 Single, incorporated, and sequential mediation effects

According to the proposed theoretical model, the authors developed hypotheses regarding the various types of mediation paths between PTC and burnout. To investigate the hypothesized mediation effects, the researchers decomposed the total indirect effects between constructs. Table 4 shows the results of the decomposition of mediation effects through the bootstrapping technique of PLS‐SEM. According to the findings, H2a and H2b are not supported, but the rest of the hypotheses are supported.

**TABLE 4:** Decomposition of mediation effects

Hypothesis	Mediated path	Effects	*t*‐statistics	Decision
	PTC → PRC	−0.339	9.486***	
	PTC → AVO → PRC	−0.236	6.277***	
	PTC → ANX → PRC	−0.103	7.261***	
	PTC → NRC	0.293	7.193***	
	PTC → AVO → NRC	0.045	2.081*	
	PTC → ANX → NRC	0.248	6.968***	
	PTC → Burnout	0.415	7.62***	
H1a	PTC → AVO → Burnout	0.010	3.264**	Supported
H1b	PTC → ANX → Burnout	0.107	2.627*	Supported
H2a	PTC → PRC → Burnout	0.006	0.563	Not supported
H2b	PTC → NRC → Burnout	0.023	0.744	Not supported
H3a	PTC → AVO → PRC → Burnout	0.035	2.141*	Supported
H3b	PTC → ANX → NRC → Burnout	0.109	7.306***	Supported
H4a	PTC → AVO → NRC → Burnout	0.020	2.066*	Supported
H4b	PTC → ANX → PRC → Burnout	0.015	2.217*	Supported
	AVO → Burnout	0.150	2.958**	
	AVO → PRC → Burnout	0.095	2.327*	
	AVO → NRC → Burnout	0.055	2.192*	
	ANX → Burnout	0.262	8.325***	
	ANX → NRC → Burnout	0.231	8.174***	
	ANX → PRC → Burnout	0.032	2.229*	

*p < .05, **p < .01, ***p < .001.

#### 5.2.3 Multiple group analysis

To detect observable heterogeneity or, in other words, the differences in the results of path analyses for different groups based on observed characteristics, the PLS‐MGA method was employed (Hair et al., 2017b). To obtain deeper insights regarding the hypothesized associations, a series of multiple group analyses based on marital status and religion type (Christianity and Islam) were conducted. The recommended sample size calculated by G*power for each group is 126 (1−*β* = 0.8, *f*2 = 0.05), which is less than each group's size. Thus, there are no issues regarding the statistical power of the conducted PLS‐MGA analyses.

To ensure that the defined groups have true differences in their structural relationships, a measurement invariance assessment (MICOM) was conducted (Hair et al., 2017a). This assessment enabled this study to evaluate the statistical power of its tests (Hult et al., 2008). The MICOM test procedures have been adopted from Henseler et al. (2016) work, in which they developed composite models to confirm the capability of created groups in measuring the specific latent variables under different circumstances. Partial and full measurement invariances have been, respectively, established for groups based on marital status and religion type. Hence, the study can proceed with PLS‐MGA. Tables 5 and 6 present the results of PLS‐MGA, respectively, according to marital status and religion type. The findings suggest that the effect of PRC on burnout is stronger and significant for the married group, but there is no other significant difference between groups based on marital status. Moreover, we did not find any significant difference between groups based on religion type.

**TABLE 5:** Bootstrapping result of partial least squares‐multigroup analysis (PLS‐MGA) analysis according to marital status

	Married(*n* = 155)		Single(*n* = 147)			
Path	Std. beta	*t*‐statistics	Std. beta	*t*‐statistics	Path coefficient difference	*t*‐statistics
PTC → AVO	0.438	8.773***	0.327	7.264***	0.112	1.473
PTC → ANX	0.504	9.891***	0.444	8.524***	0.059	0.522
PTC → PRC	−0.029	0.951	−0.052	1.128	0.023	0.356
PTC → NRC	0.031	0.693	0.074	0.735	0.043	0.502
PTC → Burnout	0.236	3.699**	0.183	3.884**	0.054	0.661
AVO → PRC	−0.582	9.129***	−0.697	8.727***	0.114	1.41
AVO → NRC	0.103	2.165*	0.141	2.377*	0.039	0.405
AVO → Burnout	0.226	3.281**	0.307	3.683**	0.081	0.857
ANX → PRC	−0.189	2.761**	−0.252	3.166**	0.062	0.648
ANX → NRC	0.492	7.947***	0.558	8.422***	0.066	0.781
ANX → Burnout	0.193	2.857**	0.249	3.374**	0.056	0.607
PRC → Burnout	−0.342	6.411***	−0.106	2.237*	0.236	2.982**
NRC → Burnout	0.417	6.801***	0.469	7.08***	0.053	0.423

*p < .05, **p < .01, ***p < .001.

**TABLE 6:** Bootstrapping result of PLS‐MGA analysis for Muslim and Christian groups

	Muslim(*n *= 149)		Christian(*n* = 153)			
Path	Std. beta	*t*‐statistics	Std. beta	*t*‐statistics	Path coefficient difference	*t*‐statistics
PTC → AVO	0.391	5.612***	0.337	5.804***	0.055	0.681
PTC → ANX	0.496	7.663***	0.432	7.192***	0.064	0.714
PTC → PRC	−0.032	0.581	−0.041	0.679	0.009	0.136
PTC → NRC	0.031	0.519	0.096	1.16	0.066	0.701
PTC → Burnout	0.247	4.179***	0.209	3.621***	0.038	0.302
AVO → PRC	−0.623	9.272***	−0.664	9.831***	0.041	0.489
AVO → NRC	0.136	2.482*	0.127	2.318*	0.008	0.142
AVO → Burnout	0.281	4.632***	0.249	4.291***	0.033	0.464
ANX → PRC	−0.193	3.607***	−0.251	4.431***	0.058	0.571
ANX → NRC	0.497	7.841***	0.564	8.473***	0.067	0.724
ANX → Burnout	0.203	3.971***	0.265	4.311***	0.062	0.683
PRC → Burnout	−0.163	2.588*	−0.218	3.146**	0.054	0.587
NRC → Burnout	0.474	6.532***	0.452	6.178***	0.023	0.361

*p < .05, **p < .01, ***p < .001.

## 6 DISCUSSION AND CONCLUSION

### 6.1 Findings and theoretical contribution

According to attachment theorists, secure people report fewer negative emotions in times of crisis (Mikulincer & Shaver, 2016), and thus it can be concluded that the threat of COVID‐19 can create the perception of a distant and unresponsive God (higher levels of avoidant and anxious attachment) among insecure people. This is aligned with the finding of this study indicating a positive relationship between the perceived threat of COVID‐19 and both avoidant and anxious attachment. Furthermore, these results agree with most articles based on the JD‐R model revealing a negative association between job demand (perceived threat of COVID‐19 in our case) and personal resources (secure attachment to God in our case; Lee & Cho, 2020).

Kim et al. (2020) found a negative relationship with stressors (immigration distress in their case) and avoidant attachment. This finding is contradictory to the results of our study, and we think that it is because of the different scales employed for avoidant attachment. Insecure attachment is defined as a strong need for the presence of attachment figures (West & Sheldon‐Keller, 1994), and if we conceptualize avoidant attachment as an insecure type of attachment, the scale developed by Beck and McDonald (2004) would not be conceptually compatible with this type of attachment since items such as “I just do not feel a deep need to be close to God” in their developed scale testify for this claim. On the other hand, the AVO in accordance with Kirkpatrick and Shaver's (1990) conceptualization is about the unavailability of God in times of need leaving the believer with a destructive feeling of loneliness and doubts in stressful times, which is a more insecure pattern than the self‐reliance theme embedded in AGI for avoidant attachment.

Explains that avoidant attachment is about the suppression of negative emotions; however, due to the insecure nature of attachment, avoidant people feel uncomfortable toward expression of positive emotions as well. There is a similarity between avoidant and secure individuals in reporting lower levels of negative appraisals, while avoidant people, unlike secure ones, are not open to closeness and relationship maintenance (Mikulincer & Shaver, 2016). This is in accordance with the findings of this study denoting AVO has a very weak positive relationship with NRC, which reflects the suppression of negative emotions and appraisals despite of their existence for avoidant people. In addition, this study finds that there is a negative relationship between AVO and PRC, which represents the minimization of efforts for closeness to God among avoidant individuals.

On the other hand, anxious attachment is about the intensification of negative emotions instead of suppression of emotional regulation (Mikulincer & Shaver, 2016). Moreover, explains that the expression of negative emotions toward attachment figures is exacerbated for those who are anxiously attached. This study finds that there is a positive relationship between anxious attachment and NRC, and also there is a negative association between the aforementioned type of attachment and PRC, which is consistent with the disclosed literature. This implies that nurses who have such attachment should endeavor to deploy tools for dealing with such anxiety, if not it will result in more stress and burnout for them.

We have found that both anxious and avoidant attachments are positively correlated with burnout, and there is consistency with our findings and previous studies presenting evidence regarding the negative consequences of insecure attachment to God in terms of cognitive, emotional, and physical complications. These studies were discussed thoroughly in attachment to the God section.

Our findings regarding the positive association of NRC and burnout as well as the negative association of PRC and burnout can be explained through a redefinition of the JD‐R model developed by Schaufeli and Taris (2014). If God's power as a personal resource becomes appraised negatively, then it would increase the job demand, which consequently exacerbates the burnout, and if it became appraised positively, it would buffer the damaging effect of job demands.

We have found that the negative relationship between PRC and burnout was stronger in the married group and was significantly different from that in the single group. This can be explained by previous evidence suggesting that individuals with a secure attachment to romantic partners are more likely to have a secure attachment to God (Kirkpatrick & Shaver, 1990), which manifests in the form of PRC (Pargament et al., 2011), and these secure attachments as personal resources have a synergistic buffering effect on the relationship between job demand and burnout. This implies that nurses who are single can try and develop deeper religious and spiritual connections than married couples, considering that they do not have an additional source of connection of a partner as married nurses.

In multiple group analysis based on religion type, we did not find any significant differences. This finding can be explained through the similarities of these Abrahamic religions. Miner et al. (2012) elucidate that both Christians and Muslims are attached to omnipotent God, and religiosity for them is about creating a close relationship with God. In this relationship, they demand relief of distress and protection from damage, and additionally, they manifest sorrow whenever they perceive God as an unavailable source.

In sum, this study contributes significantly to the literature by integrating attachment theory and the job demands‐resources model (JD‐R) to explain the role of religion in the relationship between PTC and burnout. The investigation of hypothesized mediation effects is another contribution to the literature on organizational psychology and healthcare management.

### 6.2 Practical implications

The results of this study indicated that nurses, especially those dealing with COVID‐19, experience a higher possibility of stress due to the high demand for safety measures and psychological demands of the job, hereby, in order to protect the frontline nurses aiding in the pandemic from burnout, managerial practices should be devised in a way that increase the perceived resources and decrease the perceived demands for nurses. Secure attachment to God for religious employees can be a significant stress buffer since believers perceive God as a personal resource of power and protection (Cohen & Williamson, 1991). Thus, management practices with the purpose of securing attachment to God among religious nurses are highly recommended. Organizational psychologists should encourage and teach religious staff to use PRC in stressful situations.

Psychological and religious counselors should be deployed to assist nurses during the pandemic. Williams et al. (2020) found that treatments involving self‐hypnosis can be beneficial for securing attachment to God among individuals. Managers can initiate therapies similar to the aforementioned method in order to enhance the mental well‐being of nurses. Religion‐based psychotherapy methods such as religious cognitive–emotional therapy, which employs religious beliefs and insights in therapy (Rajaei, 2010), may be advantageous for religious staff. In addition, religious counselors can be employed in order to support believers through constructive communication. Social support from religious organizations leads believers toward the employment of PRC (Krause et al., 2001).

Practices concerning the management of diversity such as religious accommodation (Cash & Gray, 2000) and the accommodation of religious expression (Kelly, 2008) are able to create faith‐friendly workplaces (Miller & Ewest, 2015). Proper management of diversity leads to the perception of sufficient job resources among employees (Pitts, 2009).

In order to decrease the perceived job demand among nurses confronting COVID‐19, managers should implement safety interventions in order to decrease the perceived threat of COVID‐19 among frontline nurses. The provision of protective equipment for nurses should be a priority since their presence are vital in the battle against the pandemic.

Designing burnout risk profiles can be an effective preventive plan for nurses’ wellbeing. Single, younger, and less experienced nurses are more vulnerable to psychological distress, and thus special actions should be planned accordingly.

### 6.3 Limitations and recommendations for future studies

This study employed self‐report questionnaires in order to collect data for the investigation of the model, which hasits own limitations in capturing in‐depth and honest responses from participants. Future studies can conduct qualitative research comprising interviews in order to extract more accurate data from respondents.

At the time of writing this article, governments have started COVID‐19 vaccinations, with priority given to healthcare workers. Therefore, for future studies, PTC among nurses may lose its relevance. However, stressful situations will not end for healthcare workers since treating COVID‐19 patients may cause secondary traumatic stress (STS). STS occurs due to indirect exposure to trauma, which is common among healthcare workers treating suffering patients. Future studies may have interest in investigation the role of religion in STS occurrence.

Future researches can investigate the role of religion in terms of different types of attachment to God and religious coping in exposure to other stressors and their respective psychological responses within organizations.

Due to the pandemic, the employment of probability sampling methods was not feasible for the authors. Researchers can contribute to the validity of our findings by conducting probability sampling for the developed model of this study.

The interactions between job demand and resources can be best understood through estimation of their reciprocal effects (see Schaufeli & Taris, 2014), which was not feasible for this study due to employment of cross‐sectional data. Future studies could employ longitudinal data to investigate the reciprocal effects between attachment to God, religious coping, and PTC.

Due to the difficulty in collecting data, it was impossible for the authors to collect sufficient data to test all the possible observed heterogeneity. Future studies can focus on multiple group analysis explaining how observable characteristics such as gender and parental status can influence the proposed model of this study. In addition, examination of this model in other cultures and religions would reveal findings regarding the generalizability of our results.

## CONFLICT OF INTEREST

The authors declare that there are no conflict of interests.

## AUTHOR CONTRIBUTION

The first author, Benard Gisilanbe Vetbuje, contributed to the conception and design, methodology and model development, discussion, conclusion, and recommendation. Panteha Farmanesh oversaw the work and contributed to the model development methodology, critical revision for intellectual content, and the final approval of the work. Arman Soosan contributed by running the analyses and interpretation of data. All authors have agreed to be accountable for all aspects of the work.

### PEER REVIEW

The peer review history for this article is available at https://publons.com/publon/10.1002/brb3.2601.

## Data Availability

Due to the nature of this research, participants of this study did not agree for their data to be shared publicly, so supporting data are not available.
